# How to Measure the Intervention Process? An Assessment of Qualitative and Quantitative Approaches to Data Collection in the Process Evaluation of Organizational Interventions

**DOI:** 10.3389/fpsyg.2016.01380

**Published:** 2016-09-22

**Authors:** Johan S. Abildgaard, Per Ø. Saksvik, Karina Nielsen

**Affiliations:** ^1^The National Research Centre for the Working EnvironmentCopenhagen, Denmark; ^2^Department of Psychology, Norwegian University of Science and TechnologyTrondheim, Norway; ^3^Norwich Business School, University of East AngliaNorwich, UK

**Keywords:** organizational interventions, qualitative methods, quantitative methods, research methodology, mixed methods, process evaluation

## Abstract

Organizational interventions aiming at improving employee health and wellbeing have proven to be challenging to evaluate. To analyze intervention processes two methodological approaches have widely been used: quantitative (often questionnaire data), or qualitative (often interviews). Both methods are established tools, but their distinct epistemological properties enable them to illuminate different aspects of organizational interventions. In this paper, we use the quantitative and qualitative process data from an organizational intervention conducted in a national postal service, where the Intervention Process Measure questionnaire (*N* = 285) as well as an extensive interview study (*N* = 50) were used. We analyze what type of knowledge about intervention processes these two methodologies provide and discuss strengths and weaknesses as well as potentials for mixed methods evaluation methodologies.

## Introduction

The evaluation of organizational interventions targeting employee health and wellbeing has been found to be a challenging task (Murta et al., 2007). The use of process evaluation, defined as the evaluation of “individual, collective or management perceptions and actions in implementing any intervention and their influence on the overall result of the intervention.” Nytrø et al. (2000) has served to increase focus on the evaluation of the specific intervention processes and not only the outcomes. Although several evaluation frameworks (Nielsen and Abildgaard, 2013; Nielsen and Randall, 2013) have been suggested it has proven to be methodologically challenging to evaluate the processes of implementation of organizational interventions (OIs; Nielsen and Randall, 2013). Two distinct approaches to process evaluation data collection are commonly used. One is a quantitative approach where either standardized or intervention-specific questionnaire items are included in a follow-up questionnaire, and are later integrated into statistical models of implementation and effect (e.g., Nielsen et al., 2007; Nielsen and Randall, 2009, 2012). The other is the collection of qualitative data; often specifically as a supplement to quantitative data, using semi-structured interviews with employees and managers, (Dahl-Jørgensen and Saksvik, 2005; Nielsen et al., 2006), observations of intervention activities (Brannan and Oultram, 2012), or long-term field observations (Czarniawska-Joerges, 2007). Qualitative process evaluation has been used extensively to understand the context of interventions outcomes (e.g., Mikkelsen and Saksvik, 1998; Saksvik et al., 2002; Nielsen et al., 2006; Aust et al., 2010). Each data source has its methodological strengths and weaknesses and the concurrent mixed methods use of both quantitative and qualitative approaches has been proposed as a potential middle ground (Dahl-Jørgensen and Saksvik, 2005; Nielsen and Randall, 2013). Mixed methods is here defined “as a method [which] focuses on collecting, analyzing and mixing both quantitative and qualitative data in a single or series of studies. Its central premise it that the use of [both] approaches in combination provides a better understanding of research problems than either approach alone” (Creswell and Plano Clark, 2011, p. 5). Although much is written about evaluation research in general (Lipsey and Cordray, 2000; Rossi et al., 2004; Pawson, 2013), and mixed methods evaluation in general (Rallis and Rossman, 2003; Nastasi et al., 2007) the particularities and methodological considerations of using qualitative and quantitative data in mixed methods based process evaluation have been sparse (Nastasi et al., 2007), particularly concerning the specifics of evaluating OIs (Nielsen and Abildgaard, 2013). Using a case of an OI in the Danish postal service where questionnaires and semi-structured interviews were used for process evaluation data collection, we compare the epistemological properties of both methods and assess the benefits of different ways to collect process information.

The aim of the present study is to examine the type of knowledge about the intervention process that may be produced by quantitative and qualitative data and discuss how these sources best can be applied in mixed methods designs. It is hence not a study of different forms of mixed methods designs (for such literature see Nastasi et al., 2007; Teddlie and Tashakkori, 2009; Creswell and Plano Clark, 2011) but instead an assessment of the properties and potential roles of specific data sources in mixed methods OI evaluation. We employ a sequential mixed methods analysis to identify a set of factors in the quantitative data that function as an analytical framework with which we comparatively analyze the qualitative data. This approach will help us accentuate what knowledge about the intervention each data collection methods may provide, and allows us to discuss differences and similarities.

### Mixed Methods OI Evaluation

Though OI evaluation has historically focused on whether the interventions improve working conditions on quantitatively measured outcomes (Griffiths, 1999) mixed methods approaches have become a commonly chosen evaluation design. A archetypical design would be the use of surveys to measure effects of the intervention (Bambra et al., 2007; Egan et al., 2007) and a, often minor (Egan et al., 2009), degree of interviews/observation to assess the process and implementation. Though this approach will cover process and effect evaluation, researchers are advocating using more methodologically rigorous qualitative methods (Griffiths, 1999; Egan et al., 2009; Nielsen and Abildgaard, 2013), as well as more integrated mixed methods approach (Nielsen et al., 2010) to iteratively collect and analyze data from different methods to improve the assessment of the intervention process (such as Nielsen et al., 2015). Additionally, in recent years scholars have more extensively included quantitative process measures (Havermans et al., 2016) which is a further argument for the necessity increased clarity of which methods are most appropriate for different mixed methods evaluation tasks. To complement the focus on stronger mixed methods methodology in OI evaluation the present study serves to shed light on what type of knowledge of the intervention is gained from qualitative and quantitative process evaluation data.

### Quantitative Process Evaluation Data Collection

A commonly used way to quantify perceptions of intervention processes is the development and use of process evaluation scales (Havermans et al., 2016). Although generic scales to measure, for instance, managerial conduct and leadership (Carless et al., 2000) exist, the quantitative process evaluation approach focuses on developing scales to measure managerial attitudes and actions related directly to the intervention in question. Established intervention measures include the Intervention Process Measure (IPM; Randall et al., 2009) and the Healthy Change Process Inventory (Tvedt et al., 2009). Other approaches include using items to quantitatively assess certain key aspects of the intervention such as employees’ participation in activities (Füllemann et al., 2015), perceived legitimacy of a change program (Biron et al., 2010), stakeholder support (Sørensen and Holman, 2014) or degree of implementation (Eklöf and Hagberg, 2006; Hasson et al., 2014). A review of the process variables used in organizational stress management intervention evaluation showed a substantial heterogeneity in the level of measurement and the constructs that are assessed (Havermans et al., 2016).

On one hand, caution is needed when using unvalidated or tailored scales (Cox and Ferguson, 1994), on the other, using context specific measures has been recommended by Randall et al. (2009), and seems especially promising as many strongly emphasize the need to take contextual differences into account (Johns, 2001; Biron et al., 2010; Nielsen and Abildgaard, 2013; Nielsen et al., 2014).

To demonstrate the potential use of quantitative process data, we analyze the questionnaire data for psychometrically valid factors, hence identifying scales. Identifying process factors via questionnaires offers opportunities to (1) ask the entire population about the intervention process, (2) link processes to outcomes and (3) test whether the process factors are generic, e.g., that line manager support is an important process factor across a range of interventions. This will contribute to our understanding of how process questionnaires are best put to use in conducting evaluation of complex OIs, and we hence pose the following research question:

Research question 1: What information about the intervention process is gained from quantitative process evaluation?

### Qualitative Process Evaluation Data Collection

The other approach, qualitative evaluation, is based on collecting and analyzing data of a very different nature. Interviews, focus groups, logbooks observations, field notes, documents, photographs, video and audio, are all valid sources, though semi-structured interviews seems to be the conventional method used in numerous studies (Mikkelsen and Saksvik, 1998; Nielsen et al., 2006, 2007; Aust et al., 2010; Biron et al., 2010; Greasley and Edwards, 2015). The semi-structured interview, being based on a prefixed interview guide with the possibility of additional follow-up questions (Kvale, 2007) allows the researcher to cover both contextual factors and intervention implementation. Other methods of choice include logbooks of activities (Gilbert-Ouimet et al., 2011; Hasson et al., 2012), consultants’ written reports of activities (Aust et al., 2010), electronic communication (Biron et al., 2010) and workplace observations supplemented with field notes or unstructured interviews (Mikkelsen and Saksvik, 1998).

Qualitative process evaluation has often been used to explain puzzling results from quantitative effect evaluation. For instance, in Aust et al. (2010), the intervention group’s working conditions deteriorated compared to the control group. Interviews indicated this deterioration was likely caused by disappointment that the OI did not deliver the expected improvements in working conditions. Nielsen et al. (2006) demonstrated how compensatory rivalry caused one control group to improve whereas unpopular concurrent changes caused the intervention to fail in one intervention group. Greasley and Edwards (2015) used extensive qualitative interviews pre- and post-intervention to assess managerial commitment and its relation to intervention success. Studies such as these demonstrate the usefulness of qualitative methods to explain unexpected effects and advance our understanding of intervention mechanisms.

In summary, it is well established that qualitative data can shed light on novel phenomena relevant to interventions, but the type of knowledge and how it differs from quantitative methods has not yet been addressed in relation to OI projects. To assess the characteristics of the knowledge gained from conducting process evaluation interviews, we aim to analyze the same constructs identified in the quantitative analysis to make comparison possible and pose the second research question:

Research question 2: What information about the intervention process is gained from qualitative process evaluation?

By answering these two research questions we contribute to the growing and diverse literature on the use of qualitative and quantitative process evaluation data in mixed methods designs by providing conceptual clarity about the epistemological properties of both methods. As we analyze the same concepts using the same intervention with different data sources we are able to compare the contributions, strengths and weaknesses of both methods. We subsequently discuss the extent of, and limits to, data collection, and how these methods can be combined in mixed methods designs regarding OI projects specifically.

## Materials and Methods

### The Organizational Intervention

The OI used a cluster randomized design in four postal areas divided in two Regions in the Postal Service. Postal service mail carriers and their line managers participated in the intervention. The OI was implemented in a participatory fashion where activities were adapted to suit the participating employees and managers. The researchers randomized the two Regions into an initial intervention group (Region 1) and a waitlist control group (Region 2) that would implement an adapted version, based on experiences from the initial OI in Region 1. In both regions the OI focused on addressing current work environment challenges as well as improving the systems for managing the long term developments of the working conditions. The key intervention components comprised an interview and questionnaire based assessment of working conditions, a detailed evaluation of health and safety practices, a prioritization workshop, and a daylong action planning workshop. In addition, ongoing steering committee meetings were held to monitor progress of activities and make decisions regarding the OI. A detailed presentation of the intervention can be found in Nielsen et al. (2013).

### Quantitative Evaluation

#### Process Items

The process questionnaire contained 22 items based on the IPM questionnaire but tailored to the specific context as recommended by Randall et al. (2009). Response options were five point Likert-type scales ranging from “strongly disagree (1)” to “strongly agree (5).” A list of the process items can be found in **Table 1**.

**Table 1:** Exploratory factor (EFA) factor structure.

Scales and constituent items	MEAN (*SD*)	Item total correlation	Common alities	Factor loadings			
				1	2	3	4
**Line manager attitudes and actions**				
My Line manager has involved the employees in the organization level intervention (OI) process	3.11 (0.98)	0.81	0.76	0.83	0.17	0.18	0.08
My Line manager has clearly explained what the benefits of the OI were	2.95 (0.97)	0.87	0.85	0.88	0.14	0.24	0.03
My Line manager has taken responsibility for implementing the OI	3.03 (0.95)	0.89	0.87	0.89	0.19	0.20	-0.02
My Line manager has prioritized to work with the OI	2.94 (0.96)	0.86	0.83	0.88	0.16	0.18	-0.03
The Area manager has done a lot to involve the employees in the implementation of the OI	2.73 (1.00)	0.77	0.68	0.78	0.15	0.22	0.07
My Line manager has done a lot to involve the employees in the implementation of the OI	2.78 (1.03)	0.79	0.72	0.78	0.14	0.29	0.11
**Improved psychosocial work environment**			
I have gained a broader understanding of psychosocial work environment and wellbeing	3.22 (0.92)	0.74	0.74	0.29	0.24	0.77	0.09
I have changed my attitude toward how wellbeing and psychosocial work environment is to be handled in my workplace	3.11 (0.91)	0.73	0.81	0.19	0.07	0.86	0.17
I have gained more influence in relation to implementing changes	2.92 (0.85)	0.74	0.75	0.31	0.25	0.77	0.01
During the last year the dialog about wellbeing and psychosocial work environment has improved	3.00 (0.92)	0.66	0.65	0.39	0.33	0.62	-0.07
**Information about changes**				
During the last year I have received information about changes in the company	3.53 (0.82)	0.76	0.74	0.21	0.80	0.20	0.12
During the last year I have received information about the prospects for the company	3.62 (0.86)	0.78	0.80	0.10	0.86	0.20	0.10
During the last year I have received information about changes in my team	3.59 (0.78)	0.79	0.78	0.15	0.85	0.14	0.09
During the last year I have received information about the prospects for my team	3.40 (0.87)	0.72	0.71	0.22	0.80	0.15	0.05
**Need for OI**							
I think we have had a need to work on improving the psychosocial work environment and the wellbeing in my team	3.38 (0.98)	0.31	0.82	-0.07	0.03	0.06	0.90
The questionnaire from the OI has focused on the things that are important for me in my work	3.30 (0.92)	0.31	0.58	0.24	0.34	0.11	0.63

#### Statistical Analyses

The existence of district scales within the items was examined using exploratory factor (EFA) with varimax rotation (*N* = 285 response rate 89%) analysis. Several items displayed a significant (*p* < 0.05) right skewed tendency, these were included based on a visual inspection, but due to the skewness principal component analysis was chosen over maximum likelihood estimation as recommended by Fabrigar et al. (1999). The EFA analyzes followed the procedures from the original IPM development.

### Qualitative Evaluation

#### The Interviews

At least two employees from each team were interviewed, in larger teams one individual and one group interview (with three employees) was conducted. The interviews were conducted at the end of the implementation phase 3 months prior to the follow-up questionnaire and followed a semi-structured interview guide. For each work team the research team selected at random a number of informants equivalent to 10% of the work team, in case the informant was not available on the day of the interview, the next person on the personnel list was selected. In total, 22 employees in Region 1 (16 individual and 2 groups) and 28 employees in Region 2 (19 individual and 3 groups) were interviewed. The interviews were tape recorded and lasted between 45 min and 1 h. The interview guide focused on the following three major topics; the intervention program and perceptions about the OI (sample question “which changes do you see the OI has brought about?”), changes in the workplace (sample question “How have your daily work tasks and schedule changed during the last year?”) and hindering and facilitating factors in the context (sample question “Which conditions in your workplace have made it difficult to achieve positive outcomes from the OI?”).

#### Qualitative Analytical Approach

Thematic analysis (Boyatzis, 1998; Braun and Clarke, 2006) was used to analyze the interview material. To assess the difference in methodological properties between qualitative and quantitative process measures we developed a thematic framework based on the factors derived from the exploratory factor analysis. The analysis focused on what qualities of the OI the interview data could illuminate. Once the thematic factors were identified in the factor analysis, all interviews were thoroughly read through and all parts relevant to specific themes were collected, subsequently an account illustrating both the breadth and depth of each theme was produced. We aimed to identify aspects relevant for understanding the working mechanisms of the OI, the personal perceptions and narratives of the OI and in that sense produce detailed contextual accounts of the OI.

## Results

### RQ1: What Information about the Intervention Process Is Gained from Quantitative Process Evaluation?

To identify what knowledge about the intervention the process items can provide we initially conducted exploratory factor analysis to identify constructs and scales for further analysis. In order to achieve a good factorial fit, six items were excluded due to high loadings on several factors (loadings > 0.2). The data had acceptable properties for conducting factor analysis (KMO = 0.89; Bartlett’s Test of Sphericity *p* < 0.000).

The factor analysis also revealed a factor structure consisting of four factors explaining 75.4% of the variance in the data. Correlation between factors and statistics from the factor analysis is presented in **Table 2**.

**Table 2:** Descriptive statistics and inter-correlations between the scales.

Scale	*M*	*SD*	1	2	3	4
Line manager attitudes and actions	2.93	0.87	1
Improved psychosocial work environment	3.06	0.76	0.60^∗∗^	1
Information about changes	3.53	0.72	0.41^∗∗^	0.50^∗∗^	1
Need for OI	3.34	0.77	0.21^∗∗^	0.27^∗∗^	0.34^∗∗^	1

*^∗∗^*P* < 0.01.*

#### Line Manager’s Actions and Attitudes

This factor consists of consists of six items which are measuring managerial actions and attitudes supporting the intervention. Cronbach’s alpha = 0.94. This factor explained 46.39% of the variance in the data.

#### Improved Psychosocial Work Environment

This factor consists of four items and covers the exposure to the intervention as well as proximal measures for intervention mechanisms (e.g., improved dialog and understanding of psychosocial work environment). Cronbach’s alpha = 0.87. This factor explained 13.8% of the variance in the data.

#### Information about Changes

This factor includes four items focusing on having received adequate information about changes relevant to the team. Cronbach’s alpha = 0.89. This factor explained 8.23% of the variance in the data.

#### Need for OI

The last factor includes two items focusing on having received adequate information about changes relevant to the team. Inter-item Correlation = 0.31. This factor explained 6.67% of the variance in the data.

#### What Knowledge Is Gained from the Questionnaires?

To answer RQ1; reducing the quantitative process questionnaire items into four distinct factors facilitated the development distinct scales, and hence provided a shortlist of the most important aspects of implementation. The quantitative data emphasized that managerial attitudes and behaviors are of particular importance (by explaining most of the variation, and also having a particularly high internal reliability). The quantitative process questionnaire provided a reduction of the complexity of the intervention into a more manageable number of components representing different aspects of the program.

An argument for the validity of the results is the fact that factors to a large extent overlap with the results found in the original IPM validation, especially the “Line manager’s attitudes and actions” and “Improved psychosocial work environment” are comprised of a subset of items from the original IPM scales. The last factor “Need for OI” is based on two items about the work environment screening questionnaire and the intervention, and has a low inter-item correlation compared to items in the others factors likely due to the two items being targeted at different, but still somewhat related, areas of perceived need (i.e., the need for a new questionnaire, and the need for the OI in general).

It is a result that is supporting quantitative measurement of OI processes that the identified constructs are in correspondence with the general literature, which has documented the distinct role of line managers, (Nielsen, 2013), the importance of information (Mattila et al., 2006), the necessity of perceived change (Semmer, 2011; Nielsen and Randall, 2012) and needs assessment (Bartholomew et al., 1998). The EFA also provided four psychometrically valid factors for use in subsequent quantitative analysis. Observing the four scales, information about changes was by the respondents rated more positively than the others, which would indicate that employees were more positive with regards to this intervention area compared to the other factors. The fact that information about changes and improvements in work environment were clearly distinct factors likewise suggests that the perceptions of information about changes in general and perceptions about the outcome of the OI did not stem from the same underlying construct. In summary, the quantitative data identifies constructs, and a quantification of their validity, reliability and interrelatedness, which can be further applied in future studies.

### Research Question 2: What Information about the Intervention Process Is Gained from Qualitative Process Evaluation?

To assess the type of information about the process that interviews may provide we analyzed the four constructs identified in the quantitative results and compared the information to that found in the qualitative data on the same topics. We first analyzed the line manager’s actions and attitudes relating to the OI, second we looked closer at the perceptions about improvements in psychosocial work environment, third, we assessed experiences relating to information about changes in the workplace, and finally, we analyzed the experiences related to need for the OI. Quotes illustrating each theme can be found in the appendix labeled “Data Sheet 1”.

#### Line Manager’s Actions and Attitudes

When the interviewees were asked about actions and attitudes of their line manager in relation to the OI they confirmed the crucial role of line managers. They elaborated on how the actions of line managers both helped and hindered implementing the OI. A majority of employees problematized the scarcity of time and the fact that line managers often prioritized focusing on other tasks than conducting and following up on OI activities.

Interviewees expanded on this perspective and underlined the key role of line managers in making sure OI progress was taking place, and that continuous communication about the intervention process was happening. Some employees expressed positive attitudes toward management’s actions during the OI, but often commented negatively on how the line managers had problems keeping their own promises. The interviews, compared to the quantitative factor, demonstrated how these everyday aspects external to the OI affected the employees’ perceptions of how the line managers were capable of supporting the implementing the OI.

#### Improvements in Psychosocial Work Environment

Many employees experienced positive developments during the implementation of the OI, most concretely improved social relations and team climate. Others agreed on the development but were not sure if it was due to the OI. Some employees expressed disappointment with regards to having spent too much time and energy on assessment and too little on developing actions. These disappointments were linked to difficulties regarding what activities stemmed from the OI and how they related to changes in working conditions.

Some expressed a hesitance about ascribing too clear causality between the OI and the improvements that could be observed, and others commented that the OI did lead to practical improvements though not on a large scale. Many interviewees likewise commented on the OI and presented their perceptions of its working mechanisms. This demonstrates how interviews can help researchers explain why and how an OI works. For example, a clear positive factor in the interviews regarding the outcome of the intervention was, for some, a feeling of being involved and participating in the development and follow-up on activities. The clear difference to the quantitative factor is the substantial doubt and hesitance expressed by the employees with regards to intervention causality. Similarly opinions and suggestion regarding weighing of the energy spent on different components of the intervention is a parameter more easily assessed by explorative qualitative methods.

#### Information about Changes in the Workplace

When asked about information about changes in the workplace, respondents talked about several interrelated issues: information about the OI activities, problems of assigning time for information distribution and general information about changes. Regarding the OI, some respondents experienced a lack of information and hence did not know where the process was headed. One interviewee explained that information did not come about by itself, one needed to actively seek out information and another employee problematized the balancing act of having limited time to seek information.

A consistent theme in the interviews was that changes in the company on a both organizational and team level significantly affected the OI and that information about these changes was insufficient. Not only did the interviewees report several cases of restructuring of work tasks but also of layoffs. These disturbances were even seen by interviewees as being used by line and area managers as excuses for not focusing sufficiently on the implementation of the OI. A problem that was raised about concurrent projects, especially during the layoffs, was that the information and developed practices were fleeting. Several interviewees hence articulated a reluctance to commit themselves to novel projects as many had substantial previous experiences with change failure. This theme demonstrated that though employees positively rated the information regarding changes in the questionnaire, their daily experiences of lack of information and navigating in a complex organization proved difficult. Likewise the interviews highlighted that the juxtaposition of wanting more information and the cost of having to spend time on acquiring it.

#### Need for the OI

Interviewees presented a lot of statements about how they perceived the need for specific aspects of the OI such as the format of being involved, developing action plans and participating. Some experienced that there had been a need for a new way of working with screening and action planning in smaller groups, while others would have preferred that everyone was participating in the activities.

In the interviews talk about the OI was also often linked to experiences with other similar activities and how they had often been forgotten in the long run. Some excused not having had sufficient time and resources for the OI due to concurrent organizational changes such as layoffs, merging teams or changing managers.

A general assessment was that the process and outcome questionnaire used in the OI was too long but some relevant aspects were identified. Some interviewees did not remember completing the questionnaire, but they often explain that they had likely done it and since forgotten about it. A group of interviewees explained that the questionnaire is superseded by concurrent events such as managerial change.

The final theme was very different in the interviews than the two items in the questionnaire. Interviewees in the semi-structured interviews did not restrain themselves to only answering the questions regarding the need for the OI, but instead gave accounts of the contextual setting that they had to assess the need for an OI in. They expressed change fatigue and compared the OI to previous failed projects and an annual attitude surveys that suffered from a lack of follow-up. Thus, the interviews provided important information about what factors employees consider before deciding whether to commit to an OI.

#### What Knowledge Is Gained from the Interviews?

The accounts and narratives identified in the four categories have a quality of being what Geertz (1973) and others have label “thick descriptions,” meaning that it is not only the direct thoughts and actions that are covered but also a detailed description of how they fit in a social context. The mental models of how employees perceive the intervention to work in their organizational context is similarly important to uncover in order to establish what mechanisms the participants’ perceive that the OI is working through (Pawson, 2013).

In the interviews we are offered explanations of how the OI fared in the practical reality of the daily postal life with hindrances such as canceled meetings, forgotten questionnaires, and unsupportive line managers. Such information is paramount in the task of providing a detailed assessment of whether an intervention as such has failed (theory failure), or it has not been adopted adequately to have had a chance to be effective (implementation failure; Nielsen et al., 2006). It allowed us to investigate, not only the degree of implementation, but also which contextual factors have caused the OI to function as it did.

A further central quality of the interviews is that they reveal how the intervention became embedded in the larger narrative of the company and became a part of the intervention history of the company. How the intervention is seen by participants compared to previous similar projects is a key result of the interviews.

## Discussion

The aim of this paper was to examine what information about the intervention process is to be gained from quantitative (RQ1) and qualitative (RQ2) process evaluation. The results in this paper have shown that for RQ1 the EFA analysis identified four distinct factors in the data, providing a set of scales for potential further inquiry and comparison. The qualitative data assessed in RQ2 in contrast demonstrated how the intervention fit the organization, and provided colorful context specific details about the intervention.

### Integrating Qualitative and Quantitative Data

A central question in mixed methods research has been how data are combined and what role different sources play in analyses (Bryman, 2007; Johnson et al., 2007; Nastasi et al., 2007; Teddlie and Tashakkori, 2009; Creswell and Plano Clark, 2011). The relevance of using a thorough qualitative assessment of the context and perceptions as well as a quantitative assessment of implementation and proximal effect of change processes seems to intuitively speak for a methodological approach where both methods are used to approximate the details of the intervention process in question (Greene et al., 1989; Rallis and Rossman, 2003; Nastasi et al., 2007). Studies have shown the potential of mixed methods by drawing on both types of process data in combination with outcome measures to get a precise estimate of processes and effects (e.g., Mikkelsen et al., 2000; Dahl-Jørgensen and Saksvik, 2005; Nielsen et al., 2006, 2015; Aust et al., 2010; Sørensen and Holman, 2014). These studies can be seen as using a form of mixed methods, labeled by Bryman (2006) as complimentary mixed methods, which demonstrates how the use of one data type (qualitative in this case) to show depth and detail can complement and nuance the results from another data type showing breadth and representativeness (quantitative in this case). The current study, however, sheds light on specific aspects of the use of qualitative and quantitative data in mixed methods evaluations of organizational interventions.

### The Usefulness of Questionnaire Measurement in Mixed Methods Designs

The fact that the quantitative process evaluation results presented a psychometrically valid factor structure with constructs that were mirrored in the qualitative data shows speaks for the validity of this method and the validity of the following characteristics: First of all a key quality of quantitative measurement is that researchers can gain valuable information about key issues from a large proportion of the sample using few resources. If intervention outcomes are measured using pre- and post-intervention questionnaires, one should not overlook the practicality of also measuring process using questionnaire items. Compared to conducting lengthy interviews or focus groups it is convenient for respondents to also answer a number of process questions that measure key constructs known to be relevant for implementation and that can be linked to quantitative outcome evaluation (Murta et al., 2007; Semmer, 2011; Nielsen and Abildgaard, 2013). Quantitative process measurement also allows for integration of process and outcome evaluation in longitudinal, mediation/moderation models with tools such as structural equation modeling (Ullman and Bentler, 2003).

Several studies have shown that interventions do not necessarily affect the entire intervention group, or have similar effects in all subgroups (Nielsen et al., 2006; Semmer, 2011). The use of quantitative data also enables for comparison of items of implementation across different contexts or intervention instances which is a substantial quality of quantitative process evaluation data.

### Understanding the Qualities of the OI Process and Context

First and foremost the qualitative interviews provided a more detailed narrative contextual account of the themes identified in the factor analysis, which gives the reader a richer understanding of the intervention and its context than the quantitative methods. The qualitative data shed light on how organizations and their members do not exist in a historical vacuum; the intervention is compared to past activities and concurrent events. The assessment of the organizational narratives that the intervention is seen through is a central quality to provide evaluation researchers and their audiences a more nuanced understanding of the “how” and “why” of intervention processes.

Qualitative data is also central for conducting a thorough process evaluation because aspects not measured in the quantitative questionnaires are likely to be affecting the results. This was seen in quotes where the employees explained nuanced aspects of line managers actions, how line managers were focusing on other aspects, how information was somehow both needed, but not wanted badly enough to call for action. Complex aspects of organizational reality, such as these, need to be uncovered using a qualitative assessment, as quantitative methods have difficulties illuminating these aspects. Similarly the interviews reveal a substantial insecurity about which outcomes are related to which activities, a problem that is not easily assessed with the questionnaires. Identifying such problematic gaps in implementation is a key benefit of explorative qualitative assessment that helps push implementation and evaluation of OIs further.

Another issue was how employees were focused on the increasing problems of downsizing and organizational change in the postal service. Conducting interviews where questions were posed about the general state of the organization made it possible to analyze how the changes were perceived, and hence how the changes might influence the outcome of the OI.

### Implications for Mixed Methods Process Evaluation

The results from this study first of all confirm the relevance and need for application of mixed methods designs to the process evaluation of organizational interventions, as different methodological tasks are better handled by applying different methods. Though this study demonstrates that it is possible to combine data sources to a mixed methods analysis of specific constructs it also puts weight behind the argument that each method would be suboptimal on its own (Greene et al., 1989; Rallis and Rossman, 2003; Nielsen et al., 2006): It is complex to accurately rate and compare degrees of implementation and support among of groups of employees using the qualitative data, and with the quantitative data novel contextual events are difficult to assess (Rallis and Rossman, 2003).

A key aspect of intervention evaluation projects is that they are linked to time limited events (i.e., the specific OI implementation), and it hence appears that researchers often conduct entirely parallel data collection designs (examples include Saksvik et al., 2002; Nielsen et al., 2006; Aust et al., 2010) possibly due to lack of time for crossover of results and adjustment of data collection strategy. In contrast to the parallel design the results from this study suggest that there are potential benefits from sequentially harnessing methods to improve the evaluation, or even using reiterative cycles of mixed methods application (Nastasi et al., 2007). The results from quantitative analyses can be used to guide, not only qualitative analysis (as was done in this study) but also the qualitative data collection to ensure that specific aspects that have been found to be puzzling are being qualitatively uncovered (Nastasi et al., 2007; Creswell and Plano Clark, 2011). Likewise interviews can be used to guide survey development to both select items and scales or even develop tailored items based on interview content (c.f. Nielsen et al., 2014).

Knowing how to balance the utilization of an efficient separate qualitative/quantitative data collection and potentially more complex and time consuming mixed methods approaches where results from different data sources are used to inform further data collection, is not an easy task (Bryman, 2007; Mertens, 2011). The question is hence not whether or not mixed methods should be used, but instead which mixed methods design is most appropriate. Here a starting point could be to examine the program theory (Pawson, 2013) underpinning the OI and consider which aspects are most appropriately and comprehensively covered by different methods.

### Strengths and Limitations

The present study used data from an OI conducted in two regions in one company. Though this is a clear limitation of the generalizability of the results, the fit with general findings in the literature suggest that the results are still usable for other researchers. As this is a study of evaluation methods, generalizability of the concrete findings is not a key quality of the study and therefore we consider the amount of data adequate.

Another limitation is that the process data collection in the intervention is very thorough in the qualitative part and perhaps not as thorough in the quantitative where only 16 items were used to measure the process. The quantitative results presented a limited picture of the intervention, but we might be able to legitimate more complex analyses if we had included more items. The survey was conducted after the interviews and hence the adaptation of the IPM would be influenced by crucial elements of the interviews.

## Conclusion

We suggest that researchers venturing into mixed methods evaluation designs carefully consider what aspects of the intervention process should be assessed by which data collection method. Qualitative process data has the potential to tie together meaning, context and narratives of the intervention and the organization. Quantitative process data in contrast has the potential to represent a larger sample of individuals’ opinions in a cost effective manner, tie together evaluation across contexts and link process and outcome measures. Both are applicable in OI evaluation but researchers must use them wisely to harness their strengths as they have different methodological presuppositions and answer different questions.

## Author Contributions

JA and KN conducted the intervention and collected the data for the study. JA wrote the draft of the paper and conducted the qualitative and quantitative analyses. PS and KN contributed substantially to its development, refinement of the analyses, presentation and discussion of the results.

## Conflict of Interest Statement

The authors declare that the research was conducted in the absence of any commercial or financial relationships that could be construed as a potential conflict of interest.
