# Supplementary material for: How to Measure the Intervention Process? An Assessment of Qualitative and Quantitative Approaches to Data Collection in the Process Evaluation of Organizational Interventions
Source: Front Psychol. 2016 Sep 22;7:1380. doi: 10.3389/fpsyg.2016.01380 (PMC5031711; doi:10.3389/fpsyg.2016.01380)
Supplement: Supplementary file 1 [file Data_Sheet_1.DOCX]

**Appendix 1: Interview Quotes Sorted by Themes**

| **Line manager attitudes and actions**  *“The managers have the resources to spend time on the OLI but I don’t think it’s on the top of their wish list. In fact I’m not sure they are able to handle the task, or it might just be lack of interest from their side”*  (Interview Region 1).  *“I think their support has been good. I’m well aware that it has been difficult for them as they have been so short time in the teams. Perhaps it is difficult to really get to know us and assess which problem areas need attention. If every place is different, then [being a line manager] has to be stressful sometimes.”*  (Interview Region 2)  *“It’s important that [the line manager] helps us stay focused, if the discussions are sidetracked during OLI activities. But also making sure that we actually do hold the meeting, so the OLI won’t fizzle out”*  (Interview Region 1).  *“Managers should perhaps have mentioned the OLI once a week, and just explain well ‘what is this about”*(Interview region 1)  *“Interviewer: How do you think your managers support of the OLI has been*  *Respondent: it’s not an easy question, as I don’t think she has been very visible. But she did participate in the workshop. If she is here and we have a problem then of course she helps us solve it.”*  (Interview region 2)  **Improved psychosocial work environment**  *“Since we started having focus on [the social climate], I have become more tolerant, had a bit more understanding and empathy in my daily conduct”*  (Interview Region 2),  *“I think we have become better at the social stuff, but I’m not sure if it has anything to do with the [OLI]” …“So many times we have identified what our problems are, and then they placed on the [kaizen] board, but no action is taken. I’ve probably just experienced that nothing ever really comes out of it”.*  (Interview Region 1)  *“Regarding our action plans there has been some improvement, but I don’t see big changes. I have a sense that, because of this project, we have been looking at our work with fresh eyes and pondered what could be different. I don’t remember if it was related to the OLI but we did have a meeting where we sat and made all these things (mapping and action plans)… I don’t think I have gotten much out of it, but somebody else might have”*  (Interview Region 1)  *“I believe that the team meeting where [the OLI facilitator] participated and we discussed some issues in the team contributed to solving the problems in the team, especially regarding one particular employee. I’m not in a position to say if it’s [the OLI] that is the cause, but I have a feeling that, it did change things. Everyone was heard and we really recognized that ‘this is for real’. I have a feeling that it changed things a bit.”* (Interview Region 2)  *We talked about it this morning, a coworker and I. It’s not like there has been massive change. We did solve the issue of one guy having two routes with newspaper, but now we only have one of those left, so it’s of no value anymore. Apart from that I don’t see the big difference, not anything I can feel. Things simply haven’t been followed up on.*  (Interview Region 2)  *“It’s important for me to be a part of the whole process and finishing it. Being as involved as possible”.* (Interview Region 2)  **Information about changes**  *“I think that, by and large, if you just get a little bit of information about what happens. It might be that things are not going as planned, but just hearing where we are and what happens – then I’m generally quite satisfied. Of course there has to be changes, but knowing that somebody is working in this and that direction [is sufficient].”*  (Group interview Region 1)  *“I don’t think that a flyer should have been used, I think we should have it on team meetings, and perhaps every time. And we need to establish work groups, so people start working on stuff”*  (Interview Region 2)  *“You have to do something yourself, then you won’t be missing anything, instead of just sitting in the corner. If there is something you need to find out, then you have to seek information yourself”*  (Interview Region 2).  *“On the one hand I don’t think there is time for more information in the workday, but on the other hand I would have liked more information [about the OLI]. I haven sought it myself though.”*  (group interview Region 2)  *”The insecurity of saying goodbye to [the two who have been laid off], “what’s happening, are there more who will be laid off”, we actually didn’t know. We didn’t know how many were to be laid off. Actually the whole layoffs came as a surprise, so there was a period with a negative atmosphere.”*  (Interview Region 2)  *“Some feel it was strange and did not know what uses it had, others thought it was ok, so it differs. […] [the seminar] was just that one day and then “boom” everything is forgotten, its business as usual with the same hurdles.”*  (Interview Region 2 )  **Need for OLI**  *”it takes some getting used to, when suddenly 10 new people come into the building with new routes and so on. It has been a huge upheaval both for them and us.*  (Interview Region 2)  *”If everybody was sitting there you would not have much influence. But when you are a group of three-four people then you get more say, and room to get your points across. Something I think is difficult when fifteen people are sitting together.”*  (Interview Region 2)  *“Sometimes I think it would be good to have everybody at the meetings, as there is substantial difference in how people look at things, there are many different opinions”*  (Interview region 1)  *“It’s important for me to be a part of the whole process and finishing it. Being as involved as possible”.* (Interview Region 2)  *“[The OI] is discussed once in a while, but it’s not something that takes up a lot of our time. I do remember that we got the questionnaire, but I don’t remember what came out of it. Nothing has been on the Kaizen boards”*  (Interview Region 1)  *”I do remember filling out the questionnaire, but I don’t remember getting any feedback on the results, it might have been that I had that particular day off”*  (Interview Region 1)  *”This summer we got the questionnaire, […] but after that nothing happened. Things got a bit chaotic around here as the leaders were moved to other areas.”*  (Group interview Region 2)  *“I do remember having filled out the questionnaire and getting feedback on it at a team meeting. I don’t quite recall what was said, but it was in line with how I experience things are around here. Of course some things were surprising as people are very different. But then you know each other, and you know sort of where the others stand”*  (Interview Region 2) |
| --- |
